# Supplementary material for: Mapping the evolution of fertility support policies in China: A content and instrumental analysis
Source: PLoS One. 2025 Oct 9;20(10):e0332137. doi: 10.1371/journal.pone.0332137 (PMC12510515; doi:10.1371/journal.pone.0332137)
Supplement: S1 Appendix — (ZIP) [file pone.0332137.s001.zip › S1 Appendix. 226 original policy documents/90-教育部等四部门关于实施第三期学前教育行动计划的意见(FBM-CLI-4-294750).doc]

教育部等四部门关于实施第三期学前教育行动计划的意见

**发布部门：** [教育部](https://www.pkulaw.com/law/chl?Aggs.IssueDepartment=60203) [机构沿革](javascript:void(0))

**发文字号：**教基〔2017〕3号

**发布日期：**2017.04.13

**实施日期：**2017.04.13

**时效性：** [现行有效](https://www.pkulaw.com/law/chl?Aggs.TimelinessDic=01)

**效力级别：** [部门工作文件](https://www.pkulaw.com/law/chl?Aggs.EffectivenessDic=XE0303)

**法规类别：** [义务与基础教育](https://www.pkulaw.com/law/chl?Aggs.Category=09117)

教育部等四部门关于实施第三期学前教育行动计划的意见
（教基〔2017〕3号）

各省、自治区、直辖市教育厅（教委）、发展改革委、财政厅（局）、人力资源社会保障厅（局），新疆生产建设兵团教育局、发展改革委、财务局、人力资源社会保障局：
　　为贯彻落实党的十八届五中全会“发展学前教育，鼓励普惠性幼儿园发展”的要求，进一步推进学前教育改革发展，经国家教育体制改革领导小组会议通过，决定2017-2020年实施第三期学前教育行动计划（以下简称三期行动计划）。现提出如下意见：
　　**一、**重要意义
　　近年来，各地按照党中央、国务院的决策部署，以县为单位实施第一期、第二期学前教育三年行动计划。各级政府高度重视，财政投入持续增加，长期制约改革发展的一些瓶颈问题得到突破。全国学前三年毛入园率2016年达到77.4%，“入园难”进一步缓解，学前教育发展迈上新的台阶。但总体上看，学前教育仍是教育体系中最薄弱的环节，普惠性资源供给不足，教师数量短缺、工资待遇偏低，幼儿园运转困难，保教质量参差不齐等问题还普遍存在，仍处于爬坡过坎的关键期。
　　学前教育作为国民教育体系的重要组成部分，对人的终身学习和发展具有重要意义。实施三期行动计划，是巩固一期二期成果，加快发展学前教育，推进教育现代化的必然要求；是基本解决“入园难”“入园贵”问题，推动两孩政策落地，保障民生的迫切需要；是推进教育扶贫，从人生早期阻断贫困代际传递，促进全面建成小康社会的重大举措。各地要深刻认识实施三期行动计划的重要意义，保持学前教育的良好发展势头，切实履职尽责，坚定不移，持续推进学前教育改革发展，努力回应人民群众对接受良好学前教育的期盼。

　　**二、**总体要求
　　（一）基本原则
　　注重科学规划。充分考虑人口政策调整和城镇化进程的需要，优化幼儿园布局。重点支持贫困地区、困难群体和薄弱环节，保障大多数适龄儿童就近接受学前教育，着力保基本、补短板、促公平。
　　坚持公益普惠。公办民办并举，进一步提高公办幼儿园提供普惠性学前教育服务的能力，积极引导和扶持民办幼儿园提供普惠性服务。加大财政投入，提升学前教育公共服务水平。
　　强化机制建设。落实地方政府发展和监管学前教育的责任，建立健全确保学前教育可持续发展的体制机制，提高综合治理能力。充分发挥中央支持政策的引导和激励作用。
　　（二）主要目标
　　到2020年，基本建成广覆盖、保基本、有质量的学前教育公共服务体系。全国学前三年毛入园率达到85%，普惠性幼儿园覆盖率（公办幼儿园和普惠性民办幼儿园在园幼儿数占在园幼儿总数的比例）达到80%左右。管理体制和办园体制逐步理顺，发展学前教育的责任进一步落实。学前教育成本分担机制普遍建立，运行保障能力显著增强。幼儿园教师配备和工资待遇保障机制初步建立，师资力量进一步加强。幼儿园保教质量评估监管体系基本形成，办园行为普遍规范，“小学化”现象基本消除。

　　**三、**重点任务
　　（一）增加普惠性资源供给。重点加强脱贫攻坚地区、两孩政策新增人口集中地区和城乡结合部幼儿园建设。大力发展公办幼儿园，提供广覆盖、保基本的学前教育公共服务。积极鼓励社会力量举办幼儿园，扶持普惠性民办幼儿园。改善办园条件，满足基本保育教育活动需要。
　　（二）深化体制机制改革。落实地方各级政府发展学前教育的责任。理顺机关、企事业单位、城镇街道办幼儿园办园体制。建立与公益普惠要求相适应的学前教育成本分担机制。深化幼儿园教师培养培训机制、补充机制和工资待遇保障机制改革。
　　（三）提升保育教育质量。深化幼儿园教育改革，坚持正确的办园方向，尊重幼儿身心发展规律和学习特点，坚持以游戏为基本活动，保教并重，养成良好的品德与行为习惯，锻炼幼儿健康的体魄，激发幼儿探究兴趣，培养积极的交往与合作能力，促进幼儿身心全面和谐发展。建立健全幼儿园保教质量评估体系，推进幼儿园质量评估工作。加强学前教育教研力量，健全教研指导网络。整体提升农村幼儿园教育质量。

　　**四、**政策措施
　　（一）发展普惠性幼儿园。逐年安排新建、改扩建一批幼儿园，支持企事业单位和集体办园，扩大公办资源。老旧城区、棚户区改造和新城区、城镇小区建设要按需要配建幼儿园。开展城镇小区配套幼儿园专项整治，对未按规定建设或移交、没有办成公办园或普惠性民办幼儿园的要全面整改，2018年底前整改到位。继续办好公办乡镇中心幼儿园，充分发挥辐射指导作用，大村独立建园，小村联合办园，优先利用中小学闲置校舍进行改建。加快集中连片贫困地区乡村幼儿园建设。各省（区、市）制定普惠性民办幼儿园认定标准，逐年确定一批普惠性民办幼儿园。通过购买服务、综合奖补、减免租金、派驻公办教师、培训教师、教研指导等方式，支持普惠性民办幼儿园发展。将提供普惠性学位数量和办园质量作为奖励和支持的依据，对达不到要求的要限期整改。
　　（二）理顺学前教育管理体制和办园体制。建立健全“国务院领导，省地（市）统筹，以县为主”的学前教育管理体制。省级、地市级政府加强统筹，加大对贫困地区支持力度。落实县级政府主体责任，充分发挥乡镇政府的作用。积极推动各地理顺机关、企事业单位、城镇街道办幼儿园办园体制，实行属地化管理，通过地方政府接收、与当地优质公办园合并、政府购买服务等多种形式，确保其面向社会提供普惠性服务。2017年底前，对符合条件的幼儿园，按照《[事业单位登记管理暂行条例](https://www.pkulaw.com/chl/4086b07817fed14dbdfb.html)》和《事业单位、社会团体及企业等组织利用国有资产举办事业单位设立登记办法（试行）》完成事业单位登记。
　　（三）健全学前教育成本分担机制。各地要按照非义务教育成本分担的要求，建立起与管理体制相适应的生均拨款、收费、资助一体化的学前教育经费投入机制，保障幼儿园正常运转和稳定发展。根据幼儿园可持续发展需要和当地实际，逐步制定公办园生均拨款标准和普惠性民办园的补助标准。进一步健全资助制度，确保建档立卡等家庭经济困难幼儿优先获得资助。根据经济发展状况、办园成本和家庭经济承受能力，对公办幼儿园的保教费收费标准进行调整。
　　（四）构建幼儿园教师队伍建设支持体系。根据普及学前三年教育的要求，确定高等学校、中等师范学校学前教育专业的培养规模和层次，加大本专科层次幼儿园教师的培养力度。支持地方通过多种方式为农村和边远贫困地区培养补充合格的幼儿园教师。采取核定编制、区县统一招考管理等方式及时补充公办幼儿园教师。根据国家有关规定和当地实际情况，采取多种方式切实解决公办幼儿园非在编教师工资待遇偏低问题，逐步实现同工同酬。引导和监督民办幼儿园依法配足配齐教职工并保障其工资待遇。幼儿园教职工依法全员纳入社保体系。到2020年，基本实现幼儿园教师全员持证上岗。深化学前教育专业课程与教学改革，提高培养质量，强化实践能力。以需求为导向，开展新一轮幼儿园教师全员培训，提高培训的针对性和实效性。各省（区、市）不断完善和全面落实符合学前教育实际，有利于幼儿园教师专业发展的职称评聘标准。
　　（五）加强幼儿园质量监管和业务指导。教育部制定幼儿园保教质量评估指南，各省（区、市）建立完善幼儿园质量评估体系，将各类幼儿园全部纳入评估范围。落实县级政府对幼儿园和培训机构的监管责任，加大监管机构和队伍的建设力度。完善幼儿园动态监管机制，规范办园行为，强化安全管理。加强玩教具配备，为幼儿创设丰富的教育环境。深入贯彻《[幼儿园工作规程](https://www.pkulaw.com/chl/111a6266c2a99711bdfb.html)》和《3-6岁儿童学习与发展指南》，指导幼儿园教师根据幼儿的发展需要制定教育计划、指导游戏活动、安排一日生活，提高保教质量。发挥乡镇中心幼儿园的辐射作用，加强对农村学前教育的业务指导，探索农村乡镇幼儿园和村幼儿园一体化管理。健全幼儿园内部财务制度，加强幼儿园经费使用和收费行为的监管。到2020年，各省（区、市）要健全学前教育管理信息系统，加强学籍管理。鼓励有条件的幼儿园面向家长和社区开展公益性0-3岁早期教育指导。

　　**五、**组织实施
　　（一）加强组织领导。县、地市、省级政府要逐级编制三期行动计划，省级和地市级政府要加强统筹，加大对贫困地区的支持力度。要把三期行动计划的实施列入政府工作的重要议事日程和相关部门的年度任务，确保各项目标任务落到实处。
　　（二）建立投入激励机制。中央财政继续安排专项资金，支持和引导地方积极发展学前教育，重点向农村地区、贫困地区倾斜。资金分配重点与各地扩大普惠性资源、完善管理体制、健全投入机制、资助家庭经济困难儿童入园等工作的绩效挂钩。
　　（三）建立工作推进机制。各地要建立学前教育综合改革协调机制，明确教育、编制、发展改革、财政、人力资源社会保障、住建、卫生计生、残联等部门的任务，着力破解长期制约学前教育发展的体制机制问题。省一级建立专项督查机制，对三期行动计划实施过程中小区配套幼儿园建设与管理、学前教育成本分担机制、加强教师队伍建设等工作情况进行专项督查。国家建立普及学前教育督导评估制度，国务院教育督导部门制定普及学前教育督导评估办法，以县为单位对普及学前教育情况进行评估，省级为主实施，国家审核认定，并将结果向社会公布。
　　各省（区、市）三期行动计划经省级人民政府批准后，于2017年7月底前报教育部备案。

教育部
国家发展改革委
财政部
人力资源社会保障部
2017年4月13日

© 北大法宝：（[www.pkulaw.com](https://www.pkulaw.com/)) 专业提供法律信息、法学知识和法律软件领域各类解决方案。北大法宝为您提供丰富的参考资料，正式引用法规条文时请与标准文本核对。
欢迎查看所有[产品和服务](https://www.pkulaw.com/helps/2.html)。法宝快讯：[如何快速找到您需要的检索结果？](https://www.pkulaw.com/helps/70.html)[法宝](https://www.pkulaw.com/helps/69.html)V6有何新特色？

扫描二维码阅读原文

**原文链接：**<https://www.pkulaw.com/chl/570ffdc37d107a00bdfb.html>
